# A Prospective Italian Study on Baseline NS3 and NS5A Resistance to Direct-Acting Antivirals in a Real-World Setting of HIV-1/HCV Coinfected Patients and Association with Treatment Outcome

**DOI:** 10.3390/v12030269

**Published:** 2020-02-28

**Authors:** Sabrina Bagaglio, Hamid Hasson, Luca Peano, Riccardo Vercesi, Emanuela Messina, Andrea Galli, Caterina Uberti-Foppa, Giulia Morsica

**Affiliations:** 1 Division of Infectious Diseases, IRCCS, Ospedale San Raffaele, 20132 Milan, Italy; bagaglio.sabrina@hsr.it (S.B.); hasson.hamid@hsr.it (H.H.); r.vercesi93@gmail.com (R.V.); messina.emanuela@hsr.it (E.M.); galli.andrea@hsr.it (A.G.); 2 Ordine dei Medici della Valle d’Aosta, 11100 Aosta, Italy; lucapeano53@gmail.com; 3 Department of Biotechnology and Biosciences, University of Milano-Bicocca, 20126 Milan, Italy; 4 Vita-Salute San Raffaele University, 20132 Milan, Italy; uberti.caterina@hsr.it

**Keywords:** HCV resistance, genotype, HIV-1, direct acting antivirals

## Abstract

We prospectively evaluated the frequency of natural resistance-associated substitutions (RASs) in the NS3 and NS5A regions according to different HCV genotypes and their possible effect on treatment outcome in HIV-1/HCV patients treated with direct-acting antivirals (DAAs). Baseline RASs in the NS3 and NS5A domains were investigated in 62 HIV-1/HCV patients treated with DAAs: 23 patients harbored HCV-GT1a, 26 harbored GT3a, and 13 harbored GT4d. A higher occurrence of RASs was found in the NS3 domain within GT1a (13/23) than GT3a (0/26) or GT4d (2/13). With regard to treatment outcome, NS3 RASs were detected in 14/56 patients with sustained virological response (SVR) and in 1/6 non-responder (NR) patients. Occurrence of RASs of NS5A domain was lower in SVR (4/56, had RASs) than in NR (3/6, had RASs). Evaluation of RASs at baseline instead of at virological failure, especially in the NS5A domain, could positively influence the choice of new DAA combinations for the treatment of HIV-1/HCV patients.

## 1. Introduction

With the introduction of direct-acting antivirals (DAAs), highly effective regimens with a sustained virological response (SVR) rate >90% is achievable in patients with HCV infection. However, a subset of patients still experience virological failure (VF) during or after treatment.

The prevalence of pre-existing resistance-associated substitutions (RASs) has been largely investigated in HCV-GT1 and HIV-negative patients, with contrasting results on the impact of naturally occurring RASs on the treatment response. The effect of RASs has been demonstrated for protease inhibitors, as the efficacy of simeprevir is reduced in patients infected with HCV-GT1a harboring the NS3 Q80K polymorphism [1,2]. Similarly, the presence of baseline NS5A RASs has been associated with a reduced viral response in some patients depending on the NS5A inhibitor and infecting genotype [3,4,5,6]. However, information on the effect of concomitant NS3 and NS5A RASs on the response rate, especially in non-GT1, is scarce among treated patients in a real-world setting, and particularly among HIV-1-infected patients [7] for whom lower adherence and unknown drug interactions may decrease treatment efficacy [8].

This report describes the baseline frequency of RASs within the NS3 and NS5A domains according to different genotypes (GT1a, GT3a, GT4d) and treatment outcomes in a group of HIV-1/HCV coinfected patients. We also considered the pattern of RASs at VF.

## 2. Material and Methods

### 2.1. Study Group

This is part of a prospective study on HIV-1/HCV coinfected patients naïve to DAA treatment. The initial study aimed to evaluate the natural resistance to NS3 and NS5A domains on the basis of the genotype of the infecting HCV and previous treatment with pegylated-interferon (Peg-IFN) plus ribavirin (experienced patients) or not (naïve patients). Patients infected with GT2 and GT1b were excluded from the study because their numbers were very limited. Of the 100 patients who provided written informed consent, in the present study we decided to include 62 who were treated with DAAs between 2015 and 2017 according to the Italian Pharma Authority (AIFA).

Routine laboratory tests for standard care of HIV/HCV coinfection were performed according to Italian [9] and international guidelines [10]. Of the 62 HIV/HCV coinfected patients, 56 (90.3%) achieved SVR defined as HCV-RNA <12 IU/mL 12 and 24 weeks post-DAA treatment. Among the six non-responder (NR) patients, five experienced virological relapse, and one patient had viral breakthrough. 

The mutational profiles of the NS3 and NS5A regions was investigated at baseline, immediately before DAA treatment in all patients according to the study protocol, and at failure in NRs as part of routine laboratory testing. 

The degree of fibrosis was assessed by transient elastography at baseline according to the metavir score. Cirrhosis was diagnosed on the basis of transient elastography and ultrasound. The study was approved by the ethics committee of San Raffaele Hospital, Milan, Italy. 

### 2.2. Sequence Analysis of the NS3 Protease Domain and NS5A Domain 1 

**Figure 1:**
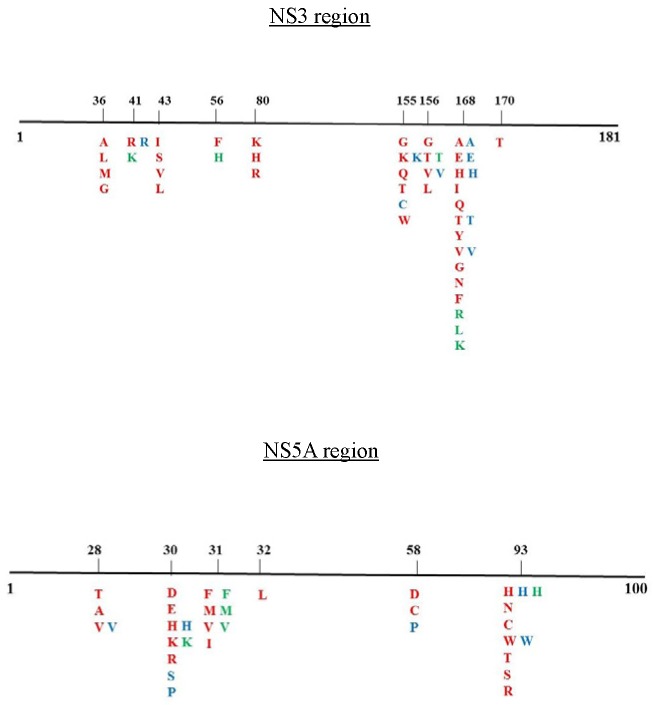
Resistance-associated substitutions identified in the NS3 protease domain (181 aa) and NS5A domain 1 (100 aa) in the literature [1,6,11]. Amino acid substitutions are color-coded based on HCV genotype: GT1a, red; GT3a, green, GT4d, blue.

The NS3 protease (181 amino acids, aa) and NS5A domain1 (100 aa) were amplified by means of nested RT-PCR using the oligonucleotides provided in Supplemental Digital Content Table S1. The PCR products were directly sequenced using an ABI 3730 XL (Applied Biosystems, Life Technologies, Italy). RAS profiles were determined from the literature [1,6,11] and by applying the Geno2Pheno HCV algorithm. A schematic representation of the RAS positions is shown in Figure 1. 

Hepatitis C genotypes determined by routine laboratory testing were confirmed by applying the Geno2Pheno (http://hcv.bioinf.mpi-inf.mpg.de) algorithm to the NS3 and NS5A sequences. The sequences were submitted to GenBank (NS3 protease accession numbers: MN650758-MN650819, NS5A accession numbers: MN641918-MN641979). 

### 2.3. Statistical Analysis

The descriptive data were presented as the number (*n*) for categorical variables, and as the median and interquartile range (IQR) for continuous variables. 

## 3. Results

### 3.1. Characteristics of HIV-1 Patients According to Presence/Absence of RASs

The characteristics of the 62 HIV-1-positive patients according to the presence/absence of RASs are summarized in Table 1. 

Overall, the majority of patients were males, had cirrhosis, a relatively preserved immune status (CD4 >250 cells/mm^3^), were virologically suppressed (HIV-1 load <50 copies/mL), and had abnormal transaminase levels. Concerning the presence/absence of RASs, the patients without RASs underwent a longer period of HIV-1 treatment and longer duration of HIV-1 infection, higher liver stiffness assessed by transient elastography, more preserved immune status (assessed by CD4 T cell count and CD4/CD8 ratio), and lower HCV-RNA viremia with respect to patients with RASs. 

### 3.2. Distribution of NS3 and NS5A RASs at Baseline 

The RAS profile according to treatment outcome (SVR or no response) is described in Table 2 and Table 3. Considering the RAS profile in the NS3 domain across GT1a, GT3a, and GT4d, we identified RASs in 15/62 sequences. NS3 RASs were detected in 13/23 GT1a isolates, and the most prominent RAS was Q80K (11/23 sequences). The GT3a isolates had no RASs in the NS3 domain, and GT4d sequences had RASs in 2/13 isolates, with D168H or Y. The NS3 RASs were detected in 7/26 IFN-R-experienced patients and 8/36 IFN-R-naïve patients. Concerning the treatment outcome, NS3 RASs were detected in 14/56 SVR patients and in 1/6 NR patients. 

Analysis of the NS5A domain across GT1a, GT3a, and GT4d revealed RASs in 7/62 sequences. The NS5A RASs were detected in 4/23 GT1a isolates, 1/26 GT3a isolates, and 2/13 GT4d isolates. Interestingly, 4/56 patients with SVR had at least one RAS, whereas 3/6 patients with VF had at least one NS5A RAS. Considering the presence of RASs along the NS3 and NS5A domains, we found that three patients had concomitant RASs in these two regions; two patients harboring GT1a (PT5 and PT10) had SVR and one patient infected by GT1a (PT58) had no response to DAA treatment. 

### 3.3. Characteristics of HIV-1/HCV Patients with Baseline RASs according to Clinical Outcome 

The RAS Q80K was detected in 10/13 GT1a sequences, and 1 GT1a-infected patient (PT5) had a double mutant (Q80K and R30P; Table 2). One other patient (PT10) had a variant with four RASs: three RASs were in the NS5A domain, and the remaining RAS was detected in the NS3 region. Two patients (PT11 and PT62) had one RAS in the NS3 region apart from Q80K. In one GT3a patient (PT43) with SVR and RAS, the mutation was located in the NS5A domain. The two patients infected by GT4d (PT9 and PT25) had exclusively NS3 RASs but were treated with sofosbuvir/ledipasvir (anti-NS5B plus anti-NS5A). Interestingly, PT36, who was treated with glecaprevir/pibrentasvir, had Y93H, which confers a high level of resistance to NS5A inhibitors, except pibrentasvir.

In six NRs, the NS3 and NS5A sequences were analyzed at failure (Table 3). In the PT58, who was infected by GT1a, the RAS Q80K in the NS3 domain and the RAS L31V plus P32R within NS5A were present before treatment, which included sofosbuvir/simeprevir (anti-NS5B and anti-NS3). At relapse, we found the double mutant Q80K/R155K, whereas the RAS L31V/P32R in NS5A disappeared.

Two patients (PT51 and PT61) infected by GT3a were treated with a sub-optimal regimen (sofosbuvir plus ribavirin) and had no RASs in the NS3 and NS5A domains at baseline or treatment failure. Three patients (PT33, PT41, PT47) infected by GT4d had no RASs in NS3 at baseline. In PT33, the NS5A RAS T58P was found at baseline, but a variant harboring D168V in the NS3 domain and T58P/Y93H in the NS5A region was found at failure. PT41 had a unique RAS (T58P in the NS5A) at both baseline and treatment failure. The remaining patient (PT47) received sub-optimal therapy with sofosbuvir plus ribavirin, with the RAS T58P in NS5A emerging at relapse.

## 4. Discussion

Despite the efficacy of DAAs, some patients still fail to eradicate HCV. Characterization of the resistance profile not only at re-treatment, but also at failure, and possibly before initiating the first DAA treatment, could be helpful in guiding treatment choice. In this context, the detection of pre-existing RASs has largely been investigated in HIV-negative patients infected with HCV-GT1, but little data are available on the presence of naturally occurring RASs in HIV-1 patients harboring GT1 or other genotypes. In the present study, we analyzed the presence of baseline NS3 and NS5A RASs according to the more representative genotypes detected in HIV-1-infected patients.

Total sequence analysis revealed RASs in 19 of 62 HIV-1 patients. Natural RASs prevailed in NS3, with more occurring in GT1a (13/23) than GT3a (0/26) and GT4d (2/13). The prevalence of naturally occurring RASs in NS3 has been found to be 11.4–40% [12,13,14,15,16,17] depending on the HCV genotype investigated and geographic origin of the isolates. One recent study [18] of HIV-1/HCV-coinfected patients reported 38% of RASs in the NS3 domain. We detected natural NS3 RASs in 15/62 (24.1%) HIV-1/HCV patients, including GT3a patients. Concerning the treatment regimen for HCV, the presence of Q80K seemed to not play a role in the response rate, as 3/4 SVR patients treated with simeprevir had the RAS Q80K, but this mutation was concomitantly detected at failure with the RAS R155K, which is also associated with resistance to simeprevir, in the unique NR patient infected with HCV-GT1a.

With regard to the RASs in NS5A, we showed that the NS5A domain was more conserved across GT1 to GT4 than the NS3 region. NS5A RASs were observed in 7/62 (11.3%) sequences, which is in line with the 6–16% reported in previous studies [19,20,21]. Carrasco et al. [6], who explored NS5A RASs across different genotypes, showed that 36.1% of their patients had NS5A RASs. We detected a lower rate of RASs, but Carrasco et al. included HIV-negative patients. In addition, they analyzed GT1b sequences, and fewer GT3a-infected patients were included than in our study. Therefore, the different patient characteristics may have contributed to the difference between our study and this previous report.

The only study to concomitantly evaluate the NS3 and NS5A RASs in HIV-1/HCV patients with acute/chronic hepatitis [7] identified RASs in 49% of patients; NS3 RASs were investigated in GT1, but not in GT3 and GT4. Among patients with chronic HCV infection, NS5A RASs were present in 13% of GT1a-infected patients, 10% of GT3a-infected patients, and 41% of GT4d-infected patients. In the present study, we found a higher number of RASs in GT1a-infected patients, but fewer RASs in GT4d-infected patients. However, the majority of GT4 patients in the previous study [7] exhibited the natural L30R polymorphism, which has not been considered as a RAS in GT4-infected patients [19,22]. Furthermore, McCormick [7] did not consider the effect of natural RASs on the treatment outcome.

Concerning SVR patients, we showed that 14/56 had RASs in the NS3 domain at baseline: four patients were treated with a simeprevir-based regimen and five were treated with DAAs, including anti-NS3 inhibitors, the activity of which is not affected by the detected variants. Five other patients had NS3 RASs but were treated with anti-NS5A and anti-NS5B inhibitors (see Table 2). Therefore, despite the high occurrence of NS3 RASs in SVR patients, only four patients had NS3 RASs that potentially reduced the viral response to the specific NS3 inhibitor (simeprevir) based regimen, including one patient (PT5) with concomitant RASs in NS3 and NS5a domains. 

Four of the 56 SVR patients had RASs in the NS5A domain: one patient was treated with anti-NS3 and anti-NS5B; one patient was treated with the anti-NS5a ledipasvir, and the detected RASs were associated with resistance to daclatasvir but not ledipasvir; one patient was treated with glecaprevir-pibrentasvir and had a RAS (Y93H) related to anti-NS5A inhibitor resistance, except pibrentasvir; and the last patient exhibited daclatasvir-specific RAS L31V, was infected by HCV GT3a, and treated with the combination of Peg-IFN-R and daclatasvir. Therefore, it is possible that the response in this patient was due to Peg-IFN-R rather than DAA treatment. 

Concerning the RAS profile in NRs, one patient infected with GT1a (PT58, treated with sofosbuvir/simeprevir) revealed an interesting pattern of RASs in NS5A; at baseline, this patient had L31V/P32R in NS5A, which disappeared at failure, when R155K emerged in NS3. We may argue that, under drug pressure, a resistant variant with a similar replication capacity as the wild-type became dominant because the strain present at baseline and harboring L31V had 40% replication activity with respect to wild-type [23].

Therefore, despite anti-NS5A inhibitors not being used in this patient, our data suggest that a future choice for re-treatment could consider sequence analysis at baseline, employing the regions not targeted by the DAA regimen. This patient was retreated with sofosbuvir/velpatasvir, the efficacy of which is not reduced by the pattern of RASs detected at baseline, exhibiting SVR. Three other patients (PT47, PT51, PT61) infected with GT4d or GT3a had no RASs, suggesting that the VF was likely due to the sub-optimal regimen (sofosbuvir plus ribavirin) employed. In one (PT33) patient treated with simeprevir plus daclatasvir, the NS5A RAS T58P was found at baseline, but at failure harbored a variant with D168V in the NS3 domain and T58P/Y93H in the NS5A domain. PT41, who received sofosbuvir plus ledipasvir, had a unique ledipasvir-associated RAS (T58P in NS5A) at baseline, as well as at treatment failure. In these two cases, the baseline RASs were specific for the compound used. 

To the best of our knowledge, this study is the first to explore the concomitant presence of natural NS3 and NS5A RASs in genotypes other than HCV-GT1 among HIV-1 infected patients, and their possible correlation with DAA treatment outcome. We found that the presence of naturally occurring RASs in NS5A seem to negatively impact the response of HIV-1/HCV patients to DAAs in a real-world setting. The higher frequency of natural RASs in the NS3 region with respect to the NS5A region, is due to the natural polymorphism Q80K being the most prominent RAS in the NS3 region, as it is exclusively found in GT1a patients. In addition, we found that this substitution had a negligible impact on the treatment outcome in our group of HIV-1/HCV patients, because 3/4 patients with SVR and receiving simeprevir had the Q80K substitution.

However, this study has some limitations. The small number of patients and the observational design of the study do not allow firm conclusions to be drawn with regard to the impact of NS5A RASs on the treatment outcome for a specific genotype. With these limitations, our data suggest that the pattern of resistance in the NS5A domain could be taken into consideration at baseline and failure, particularly in difficult-to-treat patients, to better manage re-treatment with new DAA combinations with variable efficacy according to genotype-specific substitutions.

## Figures and Tables

**Table 1:** Baseline characteristics of HIV1/HCV coinfected patients with or without resistance-associated substitutions (RASs in the NS3 and/or NS5a regions.

Variable	Category	Overall	RASs	No RASs
		*n* = 62	*n* = 19	*n* = 43
Age, years		52.0 (48.8–54.3)	51.0 (48.0–54.0)	52.0 (49.0–55.0)
Gender	female	13	2	11
	male	49	17	32
Risk factors for HIV-1 infection	sexual contact	15	7	8
	IVDU	30	6	24
	vertical transmission	1	1	0
	unknown	16	5	11
Years of HIV infection		28.0 (17.3–30.0)	24.0 (12.0–30.0)	28.0 (20.0–29.0)
Years of ART		18.0 (12.5–22.0)	16 (11.5–19.8)	18.0 (13.0–23.0)
Cirrhosis	yes	38	9	27
	no	24	6	16
Anti-HCV treatment	experienced	26	11	17
	naïve	36	8	26
Liver stiffness, KpA		17.1 (11.8–27.7)	14.6 (10.3–31.2)	17.3 (11.9–26.3)
Fibrosis	F0-F2	13	3	10
	F3-F4	49	16	33
HCV genotype	1a	23	14	9
	3a	26	1	25
	4d	13	4	9
CD4 T cells/mm^3^		634 (397–841)	524 (308–840)	642 (398–842)
Nadir CD4 T cells/mm^3^		199.5 (84.5–323.0)	199 (42–352)	200 (98–315)
CD8 T cells/mm^3^		825 (624–1155)	780 (569–1207)	827 (654–1117)
CD4/CD8 ratio		0.71 (0.45–1.13)	0.62 (0.45–1.17)	0.74 (0.43–1.14)
AST IU/L		80 (46–124)	97 (34–99)	88 (50–135)
ALT IU/L		92 (59–159)	73 (41–130)	102 (60–159)
HIV RNA < 50 copies/mL	yes	55	16	39
	no	7	3	4
Log HCV-RNA, IU/mL		6.07 (5.09–6.33)	6.17 (5.18–6.33)	5.90 (5.00–6.33)
Responded to DAA treatment	yes	56	16	40
	no	6	3	3

Characteristics were evaluated at baseline, except for the response to DAA treatment. Results are reported as median (interquartile range). IVDU = intravenous drug user, ART = antiretroviral treatment, AST = aspartate aminotransferase (normal values <35 IU/L), ALT = alanine aminotransferase (normal values <59 IU/L). Missing data: years of ART (*n* = 1); CD8 T cells/mm^3^ (*n* = 8); CD4/CD8 ratio (*n* = 8); log HCV-RNA (*n* = 6).

**Table 2:** Characteristics of 16 HIV-1/HCV coinfected patients with SVR and baseline direct-acting antivirals (DAA) resistance.

PT	Sex	Age, years	HCV GT	HCV Treatment	Fibrosis	Log HCV RNA, IU/mL	DAA (Week)	NS3 RAS	NS5A RAS
PT5	M	61	1a	experienced	F4	6.89	Sof/Sim/R (12)	Q80K	R30P
PT9	M	58	4d	experienced	F4	6.06	Sof/Ldv/R (24)	D168Y	
PT10	M	53	1a	naïve	F4	5.44	Sof/Ldv/R (24)	Q80K	K26D P32S S38C
PT11	M	53	1a	naïve	F4	6.32	Sof/Sim/R (12)	S122G	-
PT16	M	54	1a	naïve	F4	6.32	Sof/Sim/R (12)	Q80K	-
PT21	M	55	1a	naïve	F3	5.14	Ptv/r/Obv/Dsv/R (12)	Q80K	-
PT22	M	53	1a	experienced	F4	6.11	Ptv/r/Obv/Dsv/R (24)	Q80K	-
PT24 ^#^	M	50	1a	naïve	F0	-	Gzr/Ebr/R (12)	Q80K	-
PT25	M	54	4d	experienced	F3	4.75	Sof/Ldv/R (12)	D168H	-
PT30	M	50	1a	naïve	F3	6.18	Sof/Ldv/R (12)	Q80K	-
PT36	M	36	1a	naïve	F0	6.43	Gle/Pib (8)	-	Y93H
PT39	M	57	1a	experienced	F4	6.47	Sof/Sim/R (12)	Q80K	-
PT43	M	54	3a	naïve	F3	6.26	Dcv/PegIFN/R (24)	-	L31V
PT46 ^#^	M	54	1a	naïve	F2	-	Ptv/r/Obv/Dsv/R (12)	Q80K	-
PT50	M	53	1a	naïve	F3	4.54	Sof/Ldv/R (12)	Q80K	-
PT62	F	57	1a	experienced	F4	4.83	Ptv/r/Obv/Dsv/R (12)	S122G	-

PT = patient, GT = genotype, Sof = sofosbuvir, Sim = simeprevir, R = ribavirin, Ldv = ledipasvir, Ptv = paritaprevir, Obv = ombitasvir, Dsv = dasabuvir, r = ritonavir, Gzr = grazoprevir, Ebr = elbasvir, Gle = glecaprevir, Pib = pibrentasvir, Dcv = daclatasvir. ^#^ In PT24 and PT46, HCV-RNA quantitative assay was not available at baseline. - = no RASs.

**Table 3:** RAS profile in 6 HIV-1/HCV coinfected patients with no response to DAA treatment.

PT	Sex	HCV GT	HCV Treatment	Fibrosis	BL Log HCV RNA, IU/mL	DAA (Week)	BL NS3 RAS	BL NS5A RAS	FU NS3 RAS	FU NS5A RAS
PT33	F	4d	experienced	F4	6.17	Dcv/Sim/R (6)°	-	T58P	D168V	T58P Y93H
PT41	M	4d	experienced	F2	6.34	Sof/Ldv/ R (12)	-	T58P		T58P
PT47	F	4d	naïve	F4	4.96	Sof/ R (24)	-	-	-	T58P
PT51	M	3a	naïve	F4	5.81	Sof/ R (24)	-	-	-	-
PT58	M	1a	experienced	F4	5.19	Sof/Sim/R (12)	Q80K	L31V P32R	Q80K R155K	-
PT61	M	3a	naïve	F4	2.92	Sof/ R (24)	-	-	-	-

PT = patient, GT = genotype, BL = baseline, FU = follow-up, Dcv = daclatasvir, Sim = simeprevir, R = ribavirin, Sof = sofosbuvir, Ldv = ledipasvir. - = no RASs. ° In PT33 with viral breakthrough at week 6 of treatment, the Dcv/Sim association was employed on a compassionate basis.

## Supplementary Materials

Supplementary materials can be found at https://www.mdpi.com/1999-4915/12/3/269/s1.

## References

[1] Sarrazin C. (2016). The importance of resistance to direct antiviral drugs in HCV infection in clinical practice. J. Hepatol..

[2] Sorbo M.C., Cento V., Di Maio V.C., Howe A.Y.M., Garcia F., Perno C.F., Silberstein F.C. (2018). Hepatitis C virus drug resistance associated substitutions and their clinical relevance: Update 2018. Drug Resist. Updat..

[3] Fridell R.A., Wang C., Sun J.H., O’Boyle D.R., Nower P., Valera L., Qiu D., Roberts S., Huang X., Kienzle B. (2011). Genotypic and phenotypic analysis of variants resistant to hepatitis C virus nonstructural protein 5A replication complex inhibitor BMS-790052 in humans: In vitro and in vivo correlations. Hepatology.

[4] Lawitz E.J., Dvory-Sobol H., Doehle B.P., Worth A.S., McNally J., Brainard D.M., Link J.O., Miller M.D., Mo H. (2016). Clinical resistance to velpatasvir (GS-5816), a novel pan-genotypic inhibitor of the hepatitis C virus NS5A protein. Antimicrob. Agents Chemother..

[5] Krishnan P., Schnell G., Tripathi R., Beyer J., Reisch T., Dekhtyar T., Irvin M., Xie W., Fu B., Burroughs M. (2018). Integrated resistance analysis of CERTAIN-1 and CERTAIN-2 studies in hepatitis C virus-infected patients receiving glecaprevir and pibrentasvir in Japan. Antimicrob. Agents Chemother..

[6] Carrasco I., Arias A., Benítez-Gutiérrez L., Lledó G., Requena S., Cuesta M., Cuervas-Mons V., De Mendoza C. (2018). Baseline NS5A resistance associated substitutions may impair DAA response in real-world hepatitis C patients. J. Med. Virol..

[7] McCormick A.L., Moynihan L., Macartney M.J., Garcia-Diaz A., Smith C., Johnson M.A., Lumbreras C., Rubio R., Pulido F. (2015). Baseline drug resistance mutations are detectable in HCV genes NS3 and NS5A but not NS5B in acute and chronic HIV-coinfected patients. Antivir. Ther..

[8] Domínguez-Domínguez L., Bisbal O., Matarranz M., Lagarde M., Pinar Ó., Hernando A., Lumbreras C., Rubio R., Pulido F. (2019). Predictive factors of hepatitis C virus eradication after interferon-free therapy in HIV coinfection. Eur. J. Clin. Microbiol. Infect. Dis..

[9] Italian Guidelines (SIMIT). http://www.simit.org/IT/formazione/linee-guida.xhtml.

[10] International Guidelines (EACS). https://www.eacsociety.org/files/2019_guidelines-10.0_final.pdf.

[11] Lontok E., Harrington P., Howe A., Kieffer T., Lennerstrand J., Lenz O., McPhee F., Mo H., Parkin N., Pilot Matias T. (2015). Hepatitis C virus drug resistance-associated substitutions: State of the art summary. Hepatology.

[12] Chen Z.W., Li H., Ren H., Hu P. (2016). Global prevalence of pre-existing HCV variants resistant to direct-acting antiviral agents (DAAs): Mining the GenBank HCV genome data. Sci. Rep..

[13] Sarrazin C., Dvory-Sobol H., Svarovskaia E.S., Doehle B.P., Pang P.S., Chuang S.M., Ma J., Ding X., Afdhal N.H., Kowdley K.V. (2016). Prevalence of resistance-associated substitutions in HCV NS5A, NS5B, or NS3 and outcomes of treatment with ledipasvir and sofosbuvir. Gastroenterology.

[14] Bertoli A., Sorbo M.C., Aragri M., Lenci I., Teti E., Polilli E., Di Maio V.C., Gianserra L., Biliotti E., Masetti C. (2018). HCV Virology Italian Resistance Network (VIRONET-C). Prevalence of single and multiple natural NS3, NS5A and NS5B resistance-associated substitutions in hepatitis C virus genotypes 1-4 in Italy. Sci. Rep..

[15] Ehret R., Neifer S., Walter H., Baumgarten A., Obermeier M. (2014). Appearance of NS3 Q80K mutation in HCV genotype 1a mono- or HIV/HCV co-infected patients in a Berlin laboratory. J. Int. Aids Soc..

[16] Nguyen L.T., Gray E., Dean J., Carr M., Connell J., De Gascun C., Nguyen L.A., O’Leary A., Bergin C., Hall W. (2015). Baseline prevalence and emergence of protease inhibitor resistance mutations following treatment in chronic HCV genotype-1-infected individuals. Antivir. Ther..

[17] De Luca A., Di Giambenedetto S., Lo Presti A., Sierra S., Prosperi M., Cella E., Giovanetti M., Torti C., Caudai C., Vicenti I. (2015). Two distinct hepatitis C virus genotype 1a clades have different geographical distribution and association with natural resistance to NS3 protease inhibitors. Open Forum Infect. Dis..

[18] Ruggiero T., Burdino E., Calcagno A., Bonora S., Boglione L., Di Perri G., Ghisetti V. (2016). HCV NS3 naturally occurring variants in HIV/HCV coinfected DAA-naïve patients: Consideration for HCV genotyping resistance testing. Infection.

[19] Welzel T.M., Bhardwaj N., Hedskog C., Chodavarapu K., Camus G., McNally J., Brainard D., Miller M.D., Mo H., Svarovskaia E. (2017). Global epidemiology of HCV subtypes and resistance-associated substitutions evaluated by sequencing-based subtype analyses. J. Hepatol..

[20] Esposito I., Trinks J., Soriano V. (2016). Hepatitis C virus resistance to the new direct-acting antivirals. Expert Opin. Drug Metab. Toxicol..

[21] Eltahla A.A., Rodrigo C., Betz-Stablein B., Grebely J., Applegate T., Luciani F., Schinkel J., Dore G.J., Page K., Bruneau J. (2017). InC3 Study Group. Analysis of resistance-associated substitutions in acute hepatitis C virus infection by deep sequencing across six genotypes and three continents. J. Viral. Hepat..

[22] Zhou N., Hernandez D., Ueland J., Yang X., Yu F., Sims K., Yin P.D., McPhee F. (2016). NS5A sequence heterogeneity and mechanisms of daclatasvir resistance in hepatitis C virus genotype 4 Infection. J. Infect. Dis..

[23] Colson P., Gérolami R. (2011). Two years’ persistence of naturally present substitution R155K within hepatitis C virus NS3 protease in the absence of protease inhibitor-based therapy. J. Infect. Dis..

