# Supplementary material for: A Prospective Italian Study on Baseline NS3 and NS5A Resistance to Direct-Acting Antivirals in a Real-World Setting of HIV-1/HCV Coinfected Patients and Association with Treatment Outcome
Source: Viruses. 2020 Feb 28;12(3):269. doi: 10.3390/v12030269 (PMC7150799; doi:10.3390/v12030269)
Supplement: Supplementary file 1 [file viruses-12-00269-s001.pdf]

| NS3 region | | | | T annealing |
|---|---|---|---|---|
| **GT1a** | | | | |
| NS3-1a-1 | outer forward | 5′-AGCAGACAAGGGGCCTCC -3′ | (nt 3262-3281) | 50°C |
| NS3-1a-2 | outer reverse | 5′- CACCTGGAAGCTCTGGGG -3′ | (nt 4074-4093) | 50°C |
| NS3-1a-3 | inner forward | 5′-ATAATCACCAGCCTAACTGG -3′ | (nt 3288-3307) | 50°C |
| NS3-1a-4 | inner reverse | 5′-CTCATGGTTGTCTCTAGG -3′ | (nt 4038-4057) | 50°C |
| **GT3a** | | | | |
| P3A-0 | outer forward | 5′- TAATATTTAGTCCCATGGAA -3′ | (nt 3278-3297) | 50°C |
| P3A-1 | outer reverse | 5′-TATATCCTTGTGCTACATAA - 3′ | (nt 4086-4105) | 50°C |
| P3A-2 | inner forward | 5′- TCATCACCTGGGGTGCGGAT -3′ | (nt 3305-3324) | 50°C |
| P3A-3 | inner reverse | 5′- GTGCTCTTACCGCTGCCGGT -3′ | (nt 4051-4070) | 50°C |
| **GT4d** | | | | |
| F1-4 | outer forward | 5′- GGCAATGARATCYTGCTCGGSC -3′ | (nt 3263-3280) | 55°C |
| R1-4S | outer reverse | 5′- GGTCYARGACCGTGCCTATGCC -3′ | (nt 4041-4057) | 55°C |
| FIN-4 | inner forward | 5′- GGGGTGGAGRCTSCTTGCC -3′ | (nt 3287-3303) | 55°C |
| R3-4 | inner reverse | 5′- GCTCTTGCCACTTCCYGTTGG -3′ | (nt 4074-4095) | 55°C |
| **NS5A region** | | | | |
| **GT1a** | | | | |
| DOM1_1A/0 | outer forward | 5′- GAGAGCGATGCAGCTGCC -3′ | (nt 6150-6157) | 50°C |
| DOM1_1A/1 | outer reverse | 5′- CCCGTCACGTAGTGGAAGTG - 3′ | (nt 6563-6582) | 50°C |
| DOM1_1A/2 | inner forward | 5′- CTGCCATACTCAGCAGCC -3′ | (nt 6175-6192) | 50°C |
| DOM1_1A/3 | inner reverse | 5′- GCCTTATCTCCTCGTATTCC -3′ | (nt 6535-6554) | 50°C |
| **GT3a** | | | | |
| DOM1_3A/0 | outer forward | 5′- TAACTGTCACAAGTCTGCTCC -3′ | (nt 6210-6229) | 50°C |
| DOM1_3A/1 | outer reverse | 5′- CTTGGCACGGACACTTGA -3′ | (nt 6685-6702) | 50°C |
| DOM1_3A/2 | inner forward | 5′- CACCAGTGGATCAATGAAGACT -3′ | (nt 6238-6259) | 50°C |
| DOM1_3A/3 | inner reverse | 5′- TTCTGTGGCCCCCGTGAT -3′ | (nt 6661-6680) | 50°C |
| **GT4d** | | | | |
| DOM1_4/0 | outer forward | 5′- ACGCCTCCACAAGTGGAT -3′ | (nt 6153-6170) | 55°C |

| | | | | |
|---|---|---|---|---|
| DOM1_4/1 | outer reverse | 5′- GTGGGCCGGAACTTGGC -3′ | (nt 6620-6637) | 55°C |
| DOM1_4/2 | inner forward | 5′- TGCTCCACCCCATGTGCC -3′ | (nt 6181-6198) | 55°C |
| DOM1_4/3 | inner reverse | 5′- AAGTCCTCGGCGGACAC -3′ | (nt 6529-6545) | 55°C |

**Supplemental Digital Content_Table S1.** Oligonucleotides employed for GT specific amplification of the NS3 and NS5A domain.

Underlined oligonucleotides were used also for direct sequencing of PCR products .
